# Fast Determination of Rutin on a Biosensor Made Using a Layered Double Hydroxide Nanocomposite Modified Electrode

**DOI:** 10.3390/bios14010018

**Published:** 2023-12-29

**Authors:** Yuge Liu, Zhiguo Li, Weizhen Chen, Xiaomiao Feng

**Affiliations:** 1 Key Laboratory of Clean Energy Materials Chemistry of Guangdong Higher Education Institutes, School of Chemistry and Chemical Engineering, Lingnan Normal University, Zhanjiang 524048, China; lizg@lingnan.edu.cn (Z.L.); chengwz@lingnan.edu.cn (W.C.); 2 Key Laboratory for Organic Electronics and Information Displays, Institute of Advanced Materials (IAM), Nanjing University of Posts and Telecommunications, Nanjing 210023, China

**Keywords:** rutin, biosensor, LDH/graphene/polyaniline/gold, composite

## Abstract

In this study, a nanocomposite of LDH/graphene/polyaniline/gold (LDH/rGO/PANI/Au) was synthesized and characterized. The results of characterization showed that the composite material preserved the layered structure of LDH. The composite was dropped onto the glassy carbon electrode and laccase was then immobilized. Electrochemical tests showed that the composite could accelerate the electron transfer between the enzyme and the electrode. The composite/laccase showed an obvious response to rutin and the optimal detection conditions were discussed. The oxidative peak current of the biosensor constructed using the modified electrode was negatively correlated with rutin in the range of 0.05–4 μg/mL. The detection limit was 0.0017 μg/mL at a signal-to-noise ratio of 3. This biosensor of rutin also possessed high sensitivity, excellent anti-interference ability, and stability. The contents of rutin in tablets, first determined using HPLC, were also detected using the sensor constructed in this research as an application, and the results were acceptable. This research here provides a facile way for the fast detection of rutin in real samples.

## 1. Introduction

Polyphenols are ubiquitous secondary metabolites existing in most parts of a plant, including fruit, leaf, stems, root, and so on. These compounds were found to be health-profitable in scavenging reactive oxygen and nitrogen species, which may cause different kinds of disease in human beings. Flavonoids are one of the most important parts of polyphenols. They possess many properties, including antibacterial, antiviral, antitumor, anti-inflammatory, and anti-cancer. Flavonoids may also be used to treat such diseases as cardiovascular and cerebrovascular [1,2].

Rutin (3,3 4′5,7-pentahydrohyflavone-3-rhamnoglucoside, RU), with a formula of C_27_H_30_O_16_, also known as vitamin P, is a bioflavonoid glycoside existing in most plants. As an important flavonoid, the physiological functions of rutin, such as antitumor, anti-inflammatory, antibacterial, antioxidant and so on, have been extensively studied in numerous studies [3,4]. Rutin has been used to lower blood pressure and it reduces the cytotoxicity of oxidized low-density lipoprotein (LDL), as well as in clinical chemistry [5,6,7,8]. From the perspective of application, the dosage of rutin in drug tablets, food supplements, and nutraceutical inputs should be low for health and safety reasons, and it is important to establish accurate and rapid methods to determine rutin.

Up until now, some methods for rutin detecting have been extensively developed, such as a flow injection analysis [9], spectrophotometric detection [10], a fluorescence analysis [11], HPLC [12], and capillary electrophoresis [13]. Those methods possess many advantages, like high accuracy and sensitivity. However, they either need high-cost, time-consuming, expensive instruments or use toxic reagents, which limit their application. In addition, electrochemical methods were also commonly used in rutin detecting, and these methods possessed a low cost, short analysis time, and high sensitivity. There has been increasing attention on the electrochemical detection of rutin in the past few years [14,15,16,17,18,19,20,21,22,23]. Recently, Bagheri et al. manufactured a novel magnetic imprinted layer of Fe_3_O_4_@polyaniline/reduced graphene oxide molecular imprinted membrane (M@PANI/rGO MIP) on the surface of a magnetic carbon paste electrode. The modified electrode showed sensitive response to the detection of rutin with a wide linear range and ultralow detection limit (0.356 fM). The sensor fabricated was also used for the determination of rutin in fruits, and the results were almost the same as that detected through the reference method [24]. All this proved that electrochemical methods were quite effective in rutin detecting.

In the construction of biosensors for detecting rutin and other polyphenols, the addition of an enzyme (polyphenol oxidase, such as laccase) can improve the rate of catalytic reduction in polyphenols, improve the stability of the sensor and reduce the phenoxy radicals produced between a polyphenol and electrodes [25]. For biosensors, their efficiency depends not only on the method for the immobilization of the active layer on the matrix but also on the technique used for detection. Immobilization of laccase on the matrix in a rutin biosensor is quite necessary for making it a suitable catalyst, and this can also enhance the storage stability and prolong the working period of the biosensor constructed. Up until now, different kinds of materials, including carbon nanotubes, metal oxides, graphene, metal nanoparticles, and their composites, have been used as a matrix for the immobilization of enzymes in the construction of biosensors.

Among the materials used as a matrix, layered double hydroxides (LDHs), a kind of material with two-dimensional chemical structures, have attracted much attention. The general chemical formula of these materials can be depicted as [M^2+^_1−x_M^3+^_x_(OH)_2_]^x+^(An^−^)x/n·mH_2_O [26]. The structure of a typical LDH material resembles nanosheets [23]. Its key features include the presence of metal ions within the layers and anions situated between the interlayers. These kinds of materials possess many distinctive properties, such as high biocompatibility and chemical stability, pH-dependent solubility, catalytic ability, and exchange capability. Theoretically, the existence of anions between the metal interlayers permits the intercalation of any negatively charged materials with appropriate sizes. These excellent properties make LDH quite applicable in the fabrication of biosensors. Up until now, biosensors constructed using LDH modified electrodes have been widely used in food, environment, and polyphenol detection [27,28,29,30,31]. However, the small interlayer space of LDHs limits intercalation of molecules with a larger size, which restricts their application in biosensors. In addition, the low electroconductivity of LDHs also hinders their application in electrochemical fields.

Carbon materials are also widely used in biosensors. Among the multifarious carbon materials, graphene has attracted extensive attention. The exceptional properties of this material in terms of electrical, mechanical, thermal, and optical characteristics make it a significant carbon material [32]. Among the derivatives of graphene, graphene oxides (GOs) feature numerous functional groups, including hydroxyl (–OH), carbonyl (–C=O–), and carboxyl (–COOH), making them readily amenable to functionalization. After being treated through a thermal method or other ways, graphene oxides will be transformed into reduced graphene oxide (rGO). rGO possesses better electroconductibility than graphene oxides, which make it more suitable for the construction of biosensors. However, the clustering of nanosheets and the poor dispersibility in a solution of rGO hinder its application.

The combination of LDHs and GO will take the merits of both materials. On the one side, GO can greatly improve the electrical conductivity. On the other side, LDHs will enhance the chemical reactivity of GO. Therefore, it can be concluded that the resulting composites will possess extraordinary properties. In the past few years, composites of GO with different kinds of LDH have been synthesized and extensively investigated in many fields, such as drug delivery, environment protection, catalysis, energy storage, and so on [33,34,35,36,37,38,39]. Based on the excellent properties of LDHs and GO, the composite also possesses big potential in biosensors.

When preparing the composite of LDH and GO, two methods were mainly utilized. For the first method, a self-assembly process was always employed. This was based on the electrostatic attraction between LDH and GO sheets, which were positively and negatively charged, respectively. In this process, the LDHs and GO used will have to be exfoliated before preparation. If rGO is needed in the composite, GO will be reduced through the addition of a reducing agent after the precipitation of the sheets. This process can hold the layered structure of LDH. In the second method, the synthesis of an LDH/GO composite mostly comes from the in situ growth of LDH on GO in a coprecipitation process. GO can also grow inside or on the surface of LDH, which will lead to the combination of LDH/GO composites. Either method can receive composites with various properties by changing the divalent and trivalent cations during the preparation process.

**Scheme 1:**
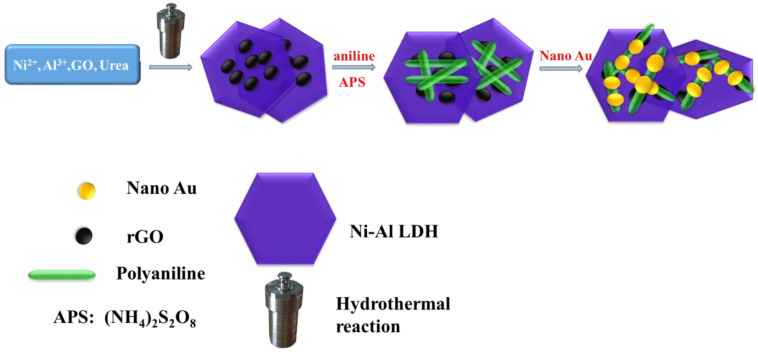
Presentation of the LDH/rGO/PANI/Au composite synthesis process.

Here in this work, a composite that contains both Ni-Al-LDH and rGO was firstly synthesized through a simple hydrothermal reaction. Different from the other methods reported, the synthesis of LDH/rGO did not need the addition of a reducing agent or the pre-exfoliation of GO and LDH. The reduction of GO occurred along with the preparation of LDH using a simple thermal treatment. Next, this material was further composited with a conductive polymer (polyaniline, PANI) and gold nanoparticles, which further improved the electroconductibility and dispersibility of the composite. The process for the synthesis of the composite is given in Scheme 1. The final material was utilized to coat a glassy carbon electrode (GCE) for laccase immobilization, and subsequently employed in rutin detection. The electrochemical sensor, created using the modified electrode, exhibited excellent electrocatalytic activity for rutin oxidation over a wide linear range with a low detection limit. The biosensor also demonstrated notable stability and anti-interference capabilities. The determination of rutin content in tablets using the sensor yielded acceptable results. This research provides an easy method for rapid rutin detection in real samples.

## 2. Experimental Section

### 2.1. Chemical Reagents

The enzyme used here, laccase (EC 1.10.3.2), was purchased from Sigma Chemical Co. (St. Louis, MI, USA) and used as received. Aniline, ammonium persulfate ((NH_4_)_2_S_2_O_8_, APS), trisodium citrate (C_6_H_5_O_7_Na_3_·2H_2_O), and HAuCl_4_ were purchased from Shanghai Chemical Reagent Company. Nickel (II) chloride, aluminum chloride, and urea were obtained from Shanghai Aladdin Biochemical Technology Co., Ltd. (Shanghai, China). Graphene oxides (GOs) were received from Nanjing XFNANO Materials Tech Co., Ltd. (Nanjing, China). The tablets of rutin were obtained from a drugstore online.

### 2.2. Synthesis of Ni-Al-LDH/rGO Composite

Synthesis of Ni-Al-LDH and Ni-Al-LDH/rGO was operated according to reports with minor changes [40,41]. The LDH synthesis closely followed established procedures. In this process, a solution comprising NiCl_2_, AlCl_3_, and urea in a molar ratio of 2:1:3 underwent a hydrothermal reaction at 180 °C. In the synthesis of LDH/rGO, the original material was the same as that of LDH, except the addition of a GO suspension to the reaction solution. Following a 48 h reaction at 180 °C, both LDH and LDH/rGO underwent filtration and were subsequently dried overnight at 40 °C.

### 2.3. Synthesis of LDH/rGO/Polymer/Au

Gold nanoparticles (NPs) were synthesized following a procedure outlined in the literature, in which a HAuCl_4_ aqueous solution was reduced using trisodium citrate [38].

For the preparation of LDH/rGO/polymer, a monomer of a polymer (aniline) was added into a solution formed by dispersing 0.1 g of LDH/rGO in 20 mL of water in the dose of 1 mmol under stirring. The stirring was continued for 30 min before the addition of an oxidizing agent (APS in 1 mol·L^−1^ HCl). The molar dosage of APS was the same as that of aniline. The reaction between the reactants continued for the following 12 h. Finally, the resulting precipitate was centrifuged and dried to obtain LDH/rGO/PANI. The electroactive composite comprising four components was achieved by immersing LDH/rGO/PANI in an Au colloid for 12 h with continuous stirring. The concentration of LDH/rGO/PANI in the mixture was 1.0 mg per 1.0 mL. The Au NPs prepared through the procedure mentioned above carry a negative charge. They can easily connect with PANI, which carries a positive charge.

### 2.4. Characterization

The morphologies of the composite and LDH were achieved through scanning electron microscopy (SEM, JSM-7610F). The powder X-ray diffraction (XRD) patterns were recorded on an X-ray diffractometer (X’Pert Powder) in the 2*θ* range of 8 to 70°. For the Fourier transform infrared (FTIR) spectroscopy of various materials, tablets with potassium bromide were first pressed and then conducted on a Fourier transform spectrometer, Nicolet 6700, and the wavelength range was from 4000 to 400 cm^−1^. The X-ray photoelectron spectroscopic (XPS) spectrum of the composite was measured on an AXIS SUPRA+ (Shimadzu, Japan) spectrometer.

### 2.5. Preparation of Electrode Modified by the Composite of LDH/rGO/PANI/Au and Electrochemical Measurements

The glassy carbon electrode (GCE) was initially polished with an alumina slurry, rinsed using water and ethanol successively, and left to dry naturally. 0.1 g of the LDH/rGO/PANI/Au composite was dispersed in 10 mL of water through ultrasonication, resulting in a solution of 2.0 mg/mL. A 5 μL portion of the resulting colloidal solution was then applied to the prepared electrode surface and allowed to dry in air. For laccase (LAC) immobilization, a solution with a concentration of 2 mg/mL was utilized. Subsequently, a Nafion solution in a mass percent of 0.5% was cast onto the modified GCE. Each step of drop-casting was followed by a 2 h drying period at ambient conditions. After these steps, laccase can be immobilized through adsorption and encapsulation [42]. 

Cyclic voltammetric and differential pulse voltammogram measurements were conducted in a standard three-electrode system on a CHI660D electrochemical workstation (Chenhua, China). In this measuring system, the glassy carbon electrode modified with LDH/rGO/PANI/Au and immobilized laccase was served as the working electrode, and an electrode of wire made of metal platinum was used as the counter electrode. For the reference electrode, a calomel electrode, filled with a saturated potassium chloride solution (SCE), was employed.

## 3. Results and Discussion

### 3.1. SEM, XRD, and FTIR Characterization

**Figure 1:**
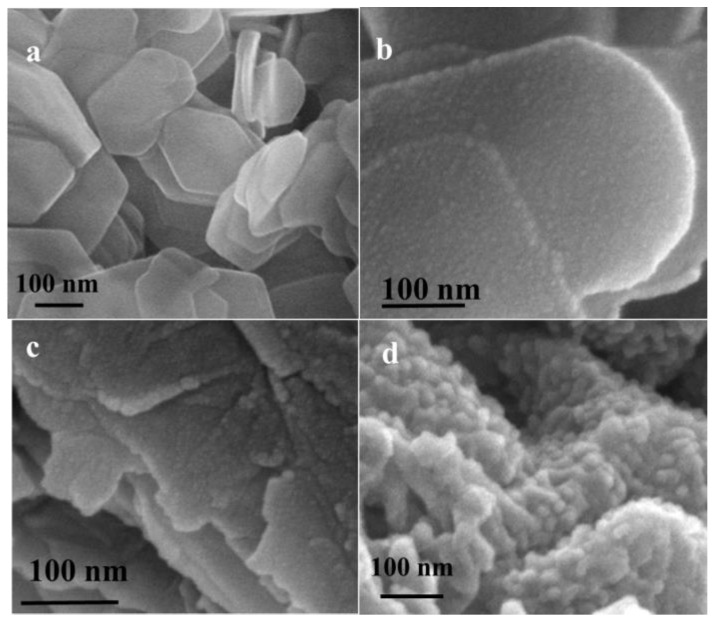
SEM images of (**a**) as-synthesized LDH, (**b**) LDH composited with reduced graphene oxides (LDH/rGO), (**c**) LDH/rGO/PANI, and (**d**) LDH/rGO/PANI/Au.

LDH obtained in the experimental section exhibited quasi-hexagonal platelet morphology, with widths ranging from 200 to 600 nm and a thickness of approximately 20 nm (Figure 1a). After being composited with rGO, the surface of the platelets became rough with small particles (Figure 1b). The particles on the surface of LDH platelets became bigger after the polymerization of aniline (Figure 1c). Finally, the diameter of nanogold on the composite was about 20 nm (Figure 1d).

**Figure 2:**
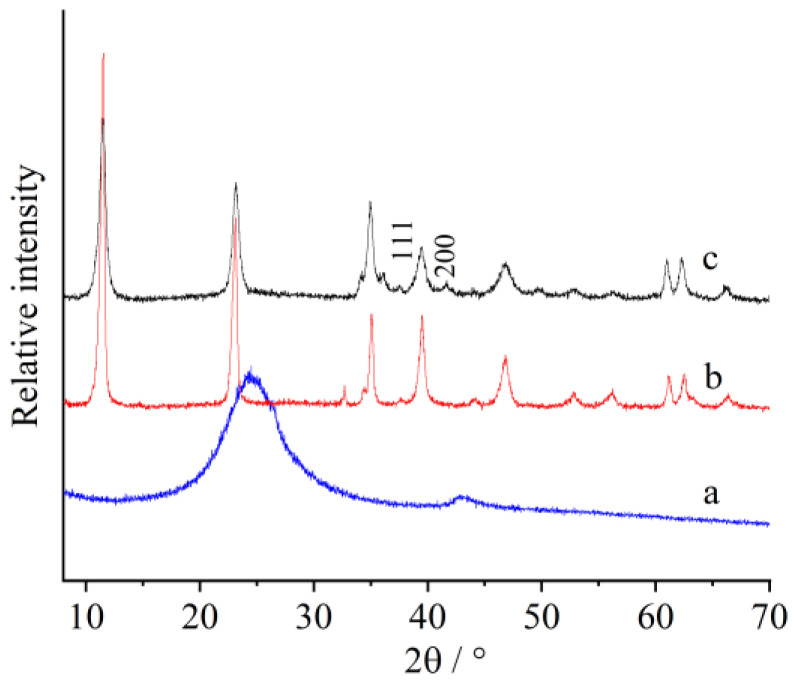
XRD patterns of (a) reduced graphene oxides (rGOs), (b) as-synthesized LDH, and (c) LDH/rGO/PANI/Au.

The XRD patterns of different materials are given in Figure 2. For rGO, there was only one broad peak at 24° (Figure 2a). The synthesized LDH showed very intense basal reflection series (Figure 2b), just the same as that reported [40]. Following the combination with rGO, PANI, and Au, two additional peaks showed up at approximately 38 and 43°. These peaks can be attributed to the (111) and (200) Bragg reflections of Au (Figure 2c). The peak of rGO disappeared in the composite, and this may be attributed to the combination with other materials.

**Figure 3:**
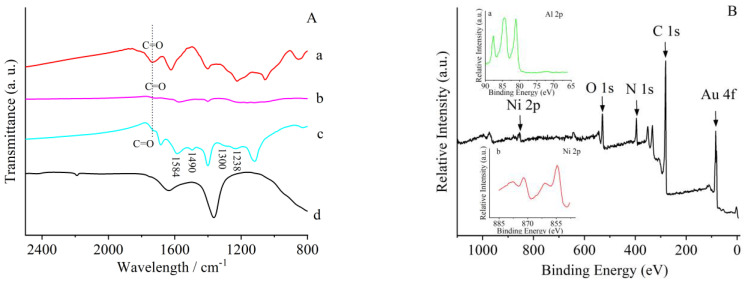
(**A**) FTIR of (a) GO, (b) reduced graphene oxides (rGOs), (c) LDH/rGO/PANI/Au, and (d) LDH. (**B**) XPS spectrum of LDH/rGO/PANI/Au; inset: enlargement of spectrum (a) Al 2p and (b) Ni 2p.

FTIR was further utilized to prove the nature of the composite. The absorption band, which was located around 1720 cm^−1^, can be identified as carboxyl groups in GO (Figure 3A(a)). This band was much smaller in rGO, indicating the reduction of GO (Figure 3A(b)) [43,44]. The same absorption band can also be seen from FTIR of the four-component composite (Figure 3A(c)). Additionally, the distinct bands at 1584 and 1490 cm^−1^ correspond to the stretching vibrations of the C=C bonds in the quinone and benzene ring, respectively [45], while peaks at 1300 and 1238 cm^−1^ represent the stretching vibrations of C-N and C=N [46]. This denoted the existence of PANI in the composite (Figure 3A(c)). The composite also possessed the typical absorption bands of LDH (Figure 3A(d)). All the above results of characterization indicated the successful preparation of the four-component LDH/rGO/PANI/Au composite.

The composite was also characterized through X-ray photoelectron spectroscopy (XPS) in a wide scan. All the elements used in the preparation of the composite can be seen in the spectrum (Figure 3B). This also illustrated the success of preparation of the composite. Compared with what has been reported, the valence state of Ni in the composite can be identified as 2 [47]. 

### 3.2. Electrochemical Measurement Condition Optimization

For the application of the composite, it was deposited onto GCE and employed for laccase (LAC) immobilization. The composite/laccase modified GCE was then utilized in the detection of rutin.

**Figure 4:**
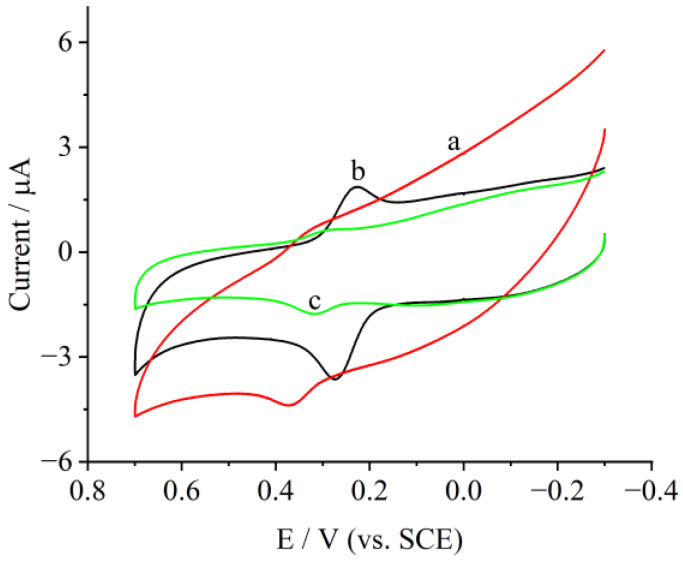
Cyclic voltammograms of LDH/rGO/PANI/Au/laccase in (a) citric acid buffer, (b) phosphate buffer, and (c) acetic acid buffer at pH 7 at 100 mV/s; the rutin concentration was 0.5 μg/mL.

To optimize the conditions for rutin biosensing, cyclic voltammograms (CVs) of the modified electrode in various pH 7 buffer solutions were firstly tested and are illustrated in Figure 4. Among the three buffers, two reversible redox peaks appeared on the modified electrode. However, the peak currents in a phosphate buffer solution (PBS) were much larger than that in both a citric acid and acetic acid buffer.

**Figure 5:**
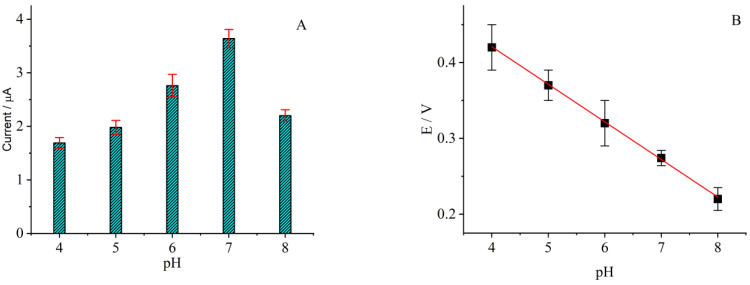
(**A**) Anodic current and (**B**) anodic potential vs. pH; the rutin concentration was 0.5 μg/mL.

PBS at different pH values was also tested and the currents of the anodic peak are shown in Figure 5A. With the increase in pH from 4 to 8, the anodic peak current initially increased and then decreased, reaching its maximum at pH 7. The anodic potential also gradually negatively shifted with an increase in pH. Within the pH range investigated here, the anodic potential was linearly related to pH, and the slope in the linear equation was 55.6 mV pH^−1^ (R^2^ = 0.99954, Figure 5B). This value was quite close to the theoretical number of 59 mV pH^−1^ at room temperature, indicating the same number of protons and electrons participated in the electrode reaction [48]. Based on the effect of the buffer and pH, PBS 7 was chosen for the rest of the electrochemical measurements.

### 3.3. Electrochemical Behavior of the Composite Modified Electrode

**Figure 6:**
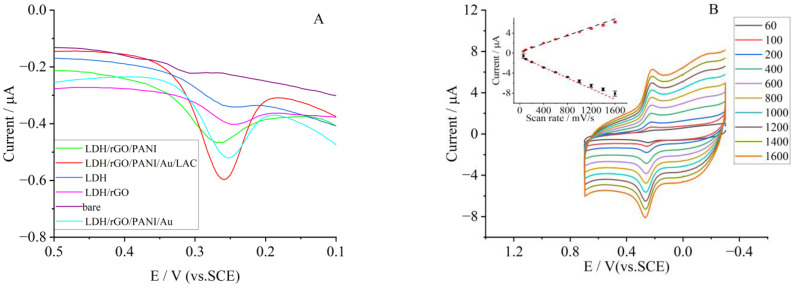
(**A**) DPV curves of different materials modified GCE in 0.1 mol·L^−1^ PBS (pH 7); the rutin concentration was 0.5 μg/mL. (**B**) CVs of LDH/rGO/PANI/Au/LAC modified electrode in 0.1 mol·L^−1^ PBS at a pH of 7.0, with a rutin concentration of 0.5 μg/mL at various scan rates. Inset: The linear relation between peak currents (anodic, black sign, and cathodic red sign) and scan rates.

The differential pulse voltammetry (DPV) of different electrodes in 0.5 μg/mL of rutin is displayed in Figure 6A. The bare GCE had a response to the oxidation of rutin. With the addition of rGO, PANI, and Au, the response gradually increased, indicating the benefit of material combination. Meanwhile, the response reached the maximum at the LDH/rGO/PANI/Au/LAC modified electrode. This indicated that laccase was quite effective in the oxidation of rutin. 

The electrode exhibits a pair of well-shaped peaks in a wide range of scan rates (*v*, mV/s), which include a low rate of 60 mV/s and a quite high rate of 1600 mV/s (Figure 6B). When the scan rate was increased, the anode peak currents (I_pa)_ of rutin linearly became bigger in a regression equation of I_pa_ = −0.0048*v* − 0.8408 (R^2^ = 0.995). The currents of the anode peak (I_pc_) were also proportional to *v* with a regression equation of I_pc_ = 0.00377*v* + 0.3828 (R^2^ = 0.996). Both the linear fitting curves are illustrated as an inset of Figure 6B. The separation between both anodic and cathodic potential follows the same pattern. All this indicates a surface-controlled process.

**Scheme 2:**
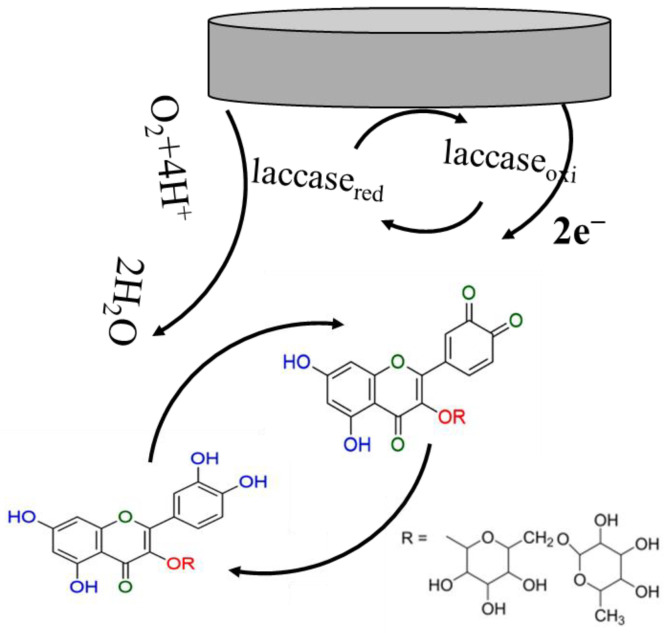
The redox of rutin on composite modified electrode surface.

In the redox process of rutin at the composite/enzyme modified electrode, the substrate was first oxidized to a free radical (semiquinone) by losing one electron, and the semiquinone would further convert into o-quinone through the loss of another electron. The resulting o-quinone will then be electrochemically reduced to rutin at the electrode surface. In the oxidation step, the electrons of rutin will be transferred by the Cu centers of laccase to the reduction of oxygen to water. During the transfer of electrons, different types of Cu of laccase were first oxidized and then reduced, serving as a catalyst of the electrochemical reaction. The catalytic mechanism of laccase was the same as that reported [49]. The redox of rutin on the modified electrode is shown in Scheme 2.

Based on the difference of ΔE_p_ and the CV of various scan rates (*v*), a linear relationship between the redox peak potentials and ln*v* was achieved. The linear regression equations between potential and ln*v* were E_pa_ (V) =0.2519 + 0.0125ln*v* (V s^−1^, R^2^ =0.993) and E_pc_ (V) = 0.2287 − 0.011ln*v* (V s^−1^, R^2^ = 0.991), respectively. According to Laviron’s equation [50], the slope of the two equations can be depicted as s RT/(1 − α)*n*F and −RT/*αn*F. Here, *α* is used for the expression of the electron-transfer coefficient, and *n* denotes the electron transfer number. With the regression equations of E_pa_ and E_pc_, *n* and α were determined to be 2.05 and 0.57, which indicated that two electrons participated in the electrode reaction.

Normally, the small value of Δ*E_p_* indicates the electron transfer rate between biomolecules and the electrode is fast, and this can be depicted by the electron transfer rate constant *k_s_*. The constant can also be calculated from Laviron’s equation as follows:

(1) $$\log k_{s}=\alpha \log(1-\alpha )+\alpha (1-\alpha )\log\alpha -\log\frac{RT}{nF\nu }-\frac{\alpha (1-\alpha )nF\Delta E_{p}}{2.3RT}$$

In this equation, *α*, *v*, and *n* possess the same meaning as described above. For R and T, they refer to the gas constant and the absolute temperature. When the absolute temperature and the scan rate was 298 K and 0.1 Vs^−1^, respectively, the constant of *k_s_* calculated using Laviron’s equation was 3.3 s^−1^. The value was double that of the electrode modified by the nanoclusters of Mg-Al-Si@PC [51]. The bigger constant of *k_s_* here indicated that the composite of LDH/rGO/PANI/Au was quite suitable for accelerating the electron transfer of the electrode reaction.

### 3.4. Determination of Rutin Using the LDH/rGO/PANI/Au/LAC Modified Electrode

**Figure 7:**
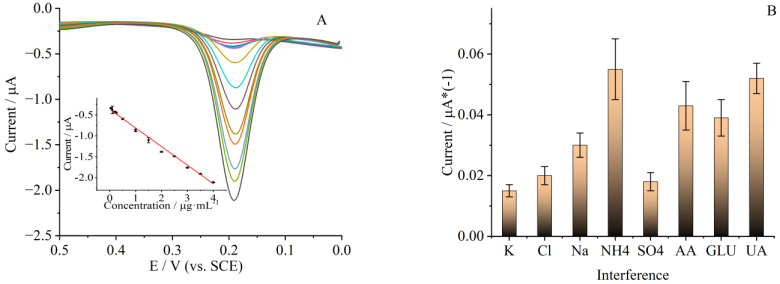
(**A**) DPV of LDH/rGO/PANI/Au/LAC modified GCE with different rutin concentrations (0.05, 0.1, 0.2, 0.25, 0.5, 1, 1.5, 2, 2.5, 3, 3.5, 4 μg/mL, from inner to outside) in 0.1 mol·L^−1^ PBS with a pH of 7. Inset: plot of linear relationship between current and rutin concentrations. (**B**) Influence of coexisting substances for rutin detection. The concentration of rutin was 0.5 μg/mL.

The electro-catalysis performance of the LDH/rGO/PANI/Au/LAC modified electrode toward rutin was evaluated using DPV, and the tests were carried out in a pH 7 PBS. The measurement results are given in Figure 7A, from which we can see that when rutin concentration was increased, the oxidation peak currents (I_pa_) would also increase gradually. A linear relationship can be achieved between I_pa_ and rutin in a concentration range of 0.05 to 4 μg/mL (0.08–6.56 μmol/L). The linear regression equation between I_pa_ and rutin concentration can be represented as follows: I_pa_ (μA) = −0.0026C (μg/mL) − 0.366. The linear correlation coefficient (R^2^) of this equation was 0.993 (inset of Figure 7A). The detection limit calculated from the regression equation was 0.0017 μg/mL (0.0028 μmol/L, S/N = 3). The sensitivity calculated from the linear regression equation and electrode surface area was 2208 μA·µmol/L· cm^−2^; this was much higher than that of the graphene nanoribbon modified electrode [16]. The higher sensitivity of this modified electrode showed that the composite was quite effective in the detection of rutin.

When the concentration of rutin in the solution exceeded 4 μg/mL, the current of oxidation first appeared as a plateau and then decreased, and this indicated a typical Michaelis–Menten kinetic mechanism. Normally, the catalytic activity of immobilized biomolecules was evaluated using the constant of Michaelis–Menten ($K_{m}^{\mathrm{app}}$), which is depicted by the Lineweaver–Burk equation [52]:

(2) $$1/I_{\mathrm{ss}}=1/I_{\max}+K_{m}^{\mathrm{app}}/I_{\max}C$$

In this equation, I_ss_ denotes the steady current when the substrate was added, and it can be achieved from the DPV response of the modified electrode after the addition of rutin. I_max_ expresses the maximum current obtained when the substrate reached its saturation. In addition, C is substrate bulk concentration. The constant calculated from the equation was 0.6 μg/mL (0.98 μmol/L) for the LDH/rGO/PANI/Au/LAC modified electrode. The small value of this constant indicated the immobilized laccase possessed a high affinity to rutin, and the enzymatic activity to the oxidation of rutin was also quite high. All this illustrated that the modified electrode here was very suitable for rutin detection.

To evaluate the electrochemical performance of the rutin biosensor constructed here, its linear range and detection limit were compared with those reported and summarized in Table 1. From the results of comparison, we can see that the linear range of the rutin biosensor in this work was reasonable, and the detection limit was lower or at the same level compared to those reported before. This may be ascribed to the excellent properties of the four components in the composite.

A sensor was constructed using the LDH/rGO/PANI/Au modified electrode, just to reveal the role of laccase. The linear range and detection limit of rutin of this sensor were from 0.4 to 2.0 μg/mL and 0.08 μg/mL, respectively. Either the linear range or the detection limit of rutin was worse than that of the LDH/rGO/PANI/Au/LAC modified electrode. This illustrated that the addition of the enzyme was quite effective in improving the performance of the biosensor in this research. 

The anti-interference performance of GCE modified by LDH/rGO/PANI/Au/LAC is quite important for accurate detection of rutin in real samples. For this purpose, DPV of the modified electrode with different interfering compounds (fixed at 10 μmol/L) and ions (100-fold of rutin concentration) was investigated and the results of oxidation peak current are given in Figure 7B. It can be seen from Figure 7B, when the potentially interfering ions (Na^+^, K^+^, Cl^−^, NH₄⁺, SO_4_^2−^), ascorbic acid (AA), glucose (GLU), and uric acid (UA) were coexisting in the solution for rutin detection, that the signal changes were quite small compared with that of rutin alone (signal change of less than 5%, n = 3).

Supplementary experiments were conducted to assess reproducibility and stability. After subjecting the system to 100 cyclic scans at 100 mV/s with a rutin concentration of 0.5 μg/mL in pH 7 PBS, no significant changes were observed in the oxidation currents between the first and the last cycle. When not used, the biosensor was stored at 4 °C. After storing for two weeks, the biosensor could still retain about 92% of its original response signal to 0.5 μg/mL in pH 7 PBS, demonstrating excellent stability. Five distinct modified GCEs were individually produced, and the associated Relative Standard Deviation (RSD) value was 4.5% for rutin determination when the concentration was 0.5 μg/mL.

For real application, rutin content in tablets was detected using the biosensor constructed above. The contents of rutin in tablets were also determined using HPLC. The results of both HPLC and the biosensor are given in Table 1, along with the recoveries. It can be concluded from Table 2 that the contents detected using the sensor were close to that of HPLC, and the recoveries were acceptable.

## 4. Conclusions

In this research, a composite of LDH/rGO/PANI/Au was synthesized. Results of characterization showed the four materials were successfully composited together. The surface of the glassy carbon electrode was decorated by the composite for application, along with the immobilization of laccase. Electrochemical results indicated that the electrode modified by the four-component composite and enzyme exhibited a noticeable response toward the oxidation of rutin, and the electron transfer rate between the electrode and enzyme was fast. For better detection of rutin, the optimal conditions for rutin detection were investigated. The biosensor constructed on the electrode modified by LDH/rGO/PANI/Au and immobilized laccase demonstrated outstanding reproducibility, stability, and anti-interference ability to the detection of rutin. The biosensor possessed a higher sensitivity and electron transfer rate constant when compared with those reported before. Additionally, it could successfully determine rutin in tablets with satisfactory results (compared with that received using HPLC). The investigation presented here offers a straightforward approach for rapid rutin determination. 

## Figures and Tables

**Table 1:** Comparison of the sensor performance with those reported before.

Modified Electrodes	Method	Linear Range (μmol L^−1^)	Detection Limit (μmol L^−1^)	References
MWCNT/CeO_2_/HRP/CPE	DPV SWV	0.5–8.0 0.2–6.0	0.3 0.16	[14]
MXene-FeWO_4_/GCE	SWV	0.004–0.147	0.00042	[15]
Gr/GNR/GCE	DPV	0.032–0.1	0.00786	[16]
AuNP-CD-LAC/CPE	SWV	0.30–2.97	0.17	[17]
laccase15/GCE	SWV	0.2–0.8 0.9–1.2	Not reported	[18]
Pt@r-O@MWCNTs/GCE	DPV	0.05–50	0.005	[20]
Mg-Al-Si@PC/GCE	DPV	1–10	0.01	[51]
PC/CoWO_4_/GCE	DPV	0.008–8	0.0074	[53]
LDH/rGO/PANI/Au/LAC/GCE	DPV	0.08–6.56	0.0028	This work

**Table 2:** Determination of rutin in tablet samples.

Sample	HPLC (mg/Tablet)	Biosensor (mg/Tablet)	Recoveries (%)	RSD
1	18	17.92 ± 0.03	99.6	4.5
2	18	18.05 ± 0.02	100.3	4.7

## Data Availability

Data are contained within the article.
